# Von Economo Neurons in the Human Medial Frontopolar Cortex

**DOI:** 10.3389/fnana.2018.00064

**Published:** 2018-08-06

**Authors:** Carlos Arturo González-Acosta, Martha Isabel Escobar, Manuel Fernando Casanova, Hernán J. Pimienta, Efraín Buriticá

**Affiliations:** ^1^Centro de Estudios Cerebrales, Facultad de Salud, Universidad del Valle, Cali, Colombia; ^2^Center for Childhood Neurotherapeutics, School of Medicine Greenville, University of South Carolina, Greenville, SC, United States

**Keywords:** von Economo neurons, pyramidal cells, fusiform cells, medial prefrontal cortex, frontopolar cortex, interhemispheric asymmetry, human cerebral cortex, area 10

## Abstract

The von Economo neurons (VEN) are characterized by a large soma, spindle-like soma, with little dendritic arborization at both, the basal and apical poles. In humans, VENs have been described in the entorhinal cortex, the hippocampal formation, the anterior cingulate cortex, the rostral portion of the insula and the dorsomedial Brodmann’s area 9 (BA9). These cortical regions have been associated with cognitive functions such as social interactions, intuition and emotional processing. Previous studies that searched for the presence of these cells in the lateral frontal poles yielded negative results. The presence of VENs in other cortical areas on the medial surface of the human prefrontal cortex which share both a common functional network and similar laminar organization, led us to examine its presence in the medial portion of the frontal pole. In the present study, we used tissue samples from five postmortem subjects taken from the polar portion of BA10, on the medial surface of both hemispheres. We found VENs in the human medial BA10, although they are very scarce and dispersed. We also observed crests and walls of the gyrus to quantitatively assess: (A) interhemispheric asymmetries, (B) the VENs/pyramidal ratio, (C) the area of the soma of VENs and (D) the difference in soma area between VENs and pyramidal and fusiform cells. We found that VENs are at least seven times more abundant on the right hemisphere and at least 2.5 times more abundant in the crest than in the walls of the gyrus. The soma size of VENs in the medial frontopolar cortex is larger than that of pyramidal and fusiform cells of layer VI, and their size is larger in the walls than in the crests. Our finding might be a contribution to the understanding of the role of these neurons in the functional networks in which all the areas in which they have been found are linked. However, the particularities of VENs in the frontal pole, as their size and quantity, may also lead us to interpret the findings in the light of other positions such as van Essen’s theory of tension-based brain morphogenesis.

## Introduction

Although some researchers, before and after von Economo, mentioned the presence of spindle-shaped neurons in diverse cortical sectors, their first characterization in humans belongs to Constantin von Economo and Georg Koskinas (von Economo and Koskinas, 1925). Today, these cells are known as von Economo neurons (VENs). In humans, VENs have been predominantly described in layer Vb of the anterior cingulate cortex, and in the anterior portion of the insula (Nimchinsky et al., 1995; Allman et al., 2011). More recently, VENs have been described in the medial prefrontal cortex, 5 mm from the dorsomedial convexity, a region corresponding to Brodmann’s area 9 (BA9; Fajardo et al., 2008). Furthermore, according to Ngowyang, VENs have been found in some sectors of the hippocampal formation (Ngowyang, 1936).

VENs have a cylindrical and elongated soma, forming a symmetrical basal and apical dendritic tree, whose branching is quite narrow as compared to adjacent pyramidal cells (Watson et al., 2006). The density of VENs in humans varies with the brain regions (Fajardo et al., 2008; Allman et al., 2011; Raghanti et al., 2015). According to volumetric analyses, it seems that they can be several times bigger than pyramidal neurons of the same cortical layer (Nimchinsky et al., 1999). Several studies have described a selective vulnerability of VENs in pathological conditions such as autism, schizophrenia, suicidal behavior and frontotemporal dementia (Allman et al., 2005; Brüne et al., 2011; Santillo et al., 2013; Uppal et al., 2014; Blanc et al., 2016; Krause et al., 2017; Yang et al., 2017; Gefen et al., 2018).

Considering aspects such as its biochemical profile, the functional role of the cortical regions that host them, its late onset in fetal development and their postnatal total number increase, several studies have even suggested that VENs could be related to emotional processing, social cognition, intuition and decision making in complex contexts (Allman et al., 2005; Cauda et al., 2013; Ibegbu et al., 2015). In addition, there are studies that suggest that these cells participate in an important network for conscious activity (Aimaretti et al., 2004; Fischer et al., 2016). However, the heterogeneity of its distribution and density in many species, in some cases with wide phylogenetic divergence, lead us to consider other hypotheses about its presumed functional role. For example, it has been proposed that its morphology constitutes a specialization of the shape of the pyramidal neurons in response to the functional demands to which they have been exposed from clades to individual species. Its greater density in the crest of the gyri (compared with the depth of the sulci) has also led to suppose that the presence of VENs could be related to the level of cortical convolution and the inherent mechanical pressure that this implies. In this way, the radial morphology of VENs and their greater density in the deep layers of the crests could be explained by the mechanical stress they experience during the process of gyrification (Van Essen, 1997).

Initially, some studies suggested that humans had the highest density of VENs compared to other non-human primates and other mammals such as cetaceans and elephants (Nimchinsky et al., 1999; Butti et al., 2009; Hakeem et al., 2009). However, a comprehensive analysis that included a wide range of non-primate species refuted this idea. That same study determined that the restricted distribution of VENs is not an exclusive phenomenon of our species (Raghanti et al., 2015).

**Figure 1:**
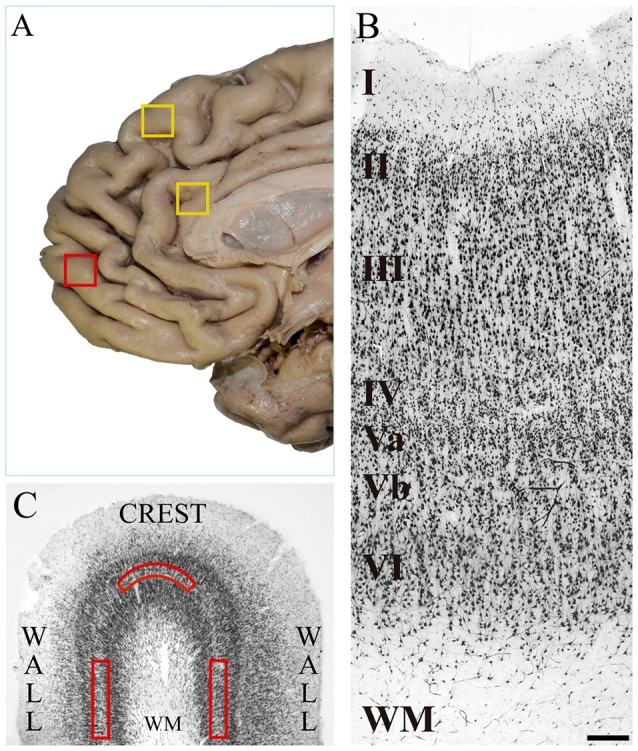
**(A)** Medial surface of the human frontal lobe. The yellow boxes show the cortical regions in which von Economo neurons (VENs) have been identified in previous studies: dorsomedial area 9 and anterior cingulate cortex. The red box shows the place where we took tissue samples in the present study, which is located on the medial surface of the frontal pole. **(B)** Microphotograph of a tissue section of human medial area 10 immunostained with anti-NeuN from the pial surface to the white matter. In the image, the cortical layers and the sublamination of layer V are indicated. The density of pyramidal cells decreases in the deeper part of layer V (Vb), and conversely, the soma area increases. Magnification 10×, scale bar = 200 μm. **(C)** Diagram of a tissue section stained with anti-NeuN showing the crest and walls of a cortical gyrus. The red boxes show the approximate location of the grids (1500 μm wide × 300 μm thick) for the neuronal counts on sublayer Vb. WM: white matter.

Our research group had previously explored several human prefrontal areas of the lateral surface (including a portion of BA10) in search of VENs with negative results (Fajardo et al., 2008). Given that the medial and orbital portions of the frontopolar cortex differ cytoarchitecturally, hodologically and functionally from the lateral portion (Öngür and Price, 2000; Buriticá and Pimienta, 2007), that VENs in humans had already been found in the anterior cingulate cortex and in the dorsomedial BA9 (see Figure 1A; yellow boxes), and that the latter presents cytoarchitecture and connections similar to those of the medial BA10, we consider plausible its location in other cortical regions of the same surface and we decided to explore the medial frontopolar cortex in search of these cells. This is the first study reporting the presence of VENs on the medial surface of the human frontopolar cortex.

## Materials and Methods

### Tissue Samples

We obtained postmortem tissue samples from five subjects from the National Institute of Forensic Medicine and Forensic Sciences (NIFMFS) in Bogotá, Colombia. The subjects (four male and one female) had ages ranging between 20 and 41 years old (mean of 25.8 years old), a postmortem interval that ranged between 8 h and 14 h (mean of 9.8 h) and the causes of death were: wound by firearm in the thorax (three cases) and wound by firearm in the spinal cord (two cases). None of them had antecedents of neurological or psychiatric diseases, brain injury or edema in the medical-forensic report. The procedures of this study were approved by the ethics committees for human research of the Faculty of Health of the Universidad del Valle and the NIFMFS, in accordance with the Helsinki protocol.

Tissue blocks (1 cm wide × 1 cm long × 1 cm deep approximately) were taken from the freshly extracted brain, washed with 10% saline solution for 5 min and then fixed by immersion. The samples were taken from the polar portion of BA10, on the medial surface of the two hemispheres, according to the cytoarchitectonic map of (Öngür et al., 2003; Buriticá and Pimienta, 2007; see Figure 1A; red box). The tissue was fixed in 4% buffered paraformaldehyde-lysine-sodium periodate, kept at a pH of 7.4 and 4°C for 7-to-10-days.

### Immunohistochemical Processing

Coronal sections (50 μm thickness) were obtained using a *Lancer Vibratome 1000 series*^®^. As a control of the correct orientation of the tissue during the sectioning process, a section every 2000 μm was stained with toluidine blue, which allowed us to verify the integrity of the tissue and the cellular orientation, that is, the presence of the entire cortex from layer I to the subcortical white matter and the vertical organization of the apical dendrites of the pyramidal neurons. Every 800 μm a section of tissue was collected to be processed by immunohistochemistry with anti-NeuN antibody. We collected between 9 and 12 tissue sections per subject to be analyzed in the present study. The remaining sections were collected for purposes other than those of the present study.

Initially, the sections were introduced into a mixture of 0.3% hydrogen peroxide and 30% absolute methanol to prevent the action of the endogenous peroxidase. To avoid adhesion to non-specific antigens, we used normal horse serum diluted 1.5% in phosphate buffered saline (PBS) for 40 min. The tissue was incubated in anti-NeuN antibody (Anti-NeuN Antibody, clone A60, MAB377 EDM Millipore-Sigma Aldrich, Merck) for 18 h. The primary antibody was diluted in PBS with 0.5% Triton X-100 (1:2500). Then, the sections were incubated in the avidin-biotin HRP complex (Vectastain Elite ABC, PK-6102 Mouse IgG; Vector Laboratories) for 1.5 h. Finally, they were processed in a solution of 4% 3,3′-diaminobenzidine, 2% hydrogen peroxide and 2% nickel in PBS (peroxidase substrate kit, DAB SK-4100; Vector Laboratories) for 5 min. After each treatment, three washes of 5 min each were carried out with PBS. With the tissue already subjected to immunostaining, it was mounted on chrome-lighted glass plates which were then washed with distilled water and dehydrated with graded alcohols and xylols. The tissues were cover-slipped with histological mounting media (Permount mounting media, Fisher Scientific, Thermo Fisher Scientific). As a negative control, tissue sections were processed in the same way previously described except that the primary antibody was omitted. All procedures were carried out at room temperature.

### Histological Observation

The histological observation of the tissue sections was performed with a light microscope ZEISS Scope.A1, coupled with a high-resolution camera AxioCam HRc. In total we had 57 tissue sections, 30 of the right hemisphere and 27 of the left hemisphere. Given the small size of the samples provided by the NIFMFS, we only had the crest and the walls of one gyrus per subject without the deep part of the sulci (see diagram of Figure 1C). After verifying the cytoarchitectonic characteristics of the medial BA10 (see Figure 1B) according to the experience of the two observers in previous studies (Buriticá and Pimienta, 2007; Arteaga et al., 2015), we proceeded to search cells with the typical morphological characteristics of VENs along the entire layer Vb of each of the tissue sections processed histologically. The search for VENs was restricted to layer Vb because the deep part of this layer is where they have been found in other human cortical regions. The cytoarchitectonic verification of the medial frontopolar cortex was made using magnifications of 4× and 10×; and the search for VENs with magnifications of 10× and 40×.

**Figure 2:**
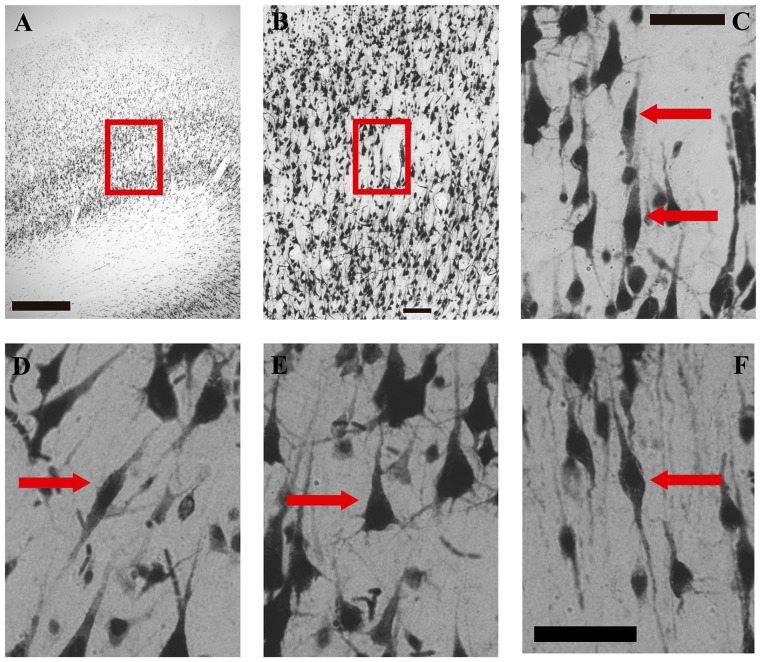
Microphotographs of VENs, pyramidal and fusiform cells in the human medial frontopolar cortex. **(A–C)** Correspond to photographs taken at different magnifications of the same sector of one of the walls of a gyrus. **(D–F)** are high-magnification photographs (40×) of a VENs and a pyramidal cell of sublayer Vb, and of a fusiform cell of layer VI. **(A)** Portion of a tissue section where VENs were found in the wall of a gyrus. The red box shows the sector located between layers IV and VI which will be enlarged on image **(B)**. Magnification 2.5×, scale bar = 500 μm. **(B)** Microphotograph illustrating a magnification of the red box of image **(A)**. Magnification 10×, scale bar = 100 μm. **(C)** Microphotograph illustrating a magnification of the red box in image **(B)**. The red arrows indicate two NeuN-positive cells with the typical morphology of VENs identified in sublayer Vb of the medial frontopolar cortex of humans. Magnification 40×, scale bar = 50 μm. **(D)** Photomicrograph illustrating a VEN of sublayer Vb indicated by the red arrow. **(E)** Microphotograph illustrating a pyramidal cell of sublayer Vb indicated by the red arrow. **(F)** Microphotograph illustrating a fusiform cell of layer VI indicated by the red arrow. Scale bar = 50 μm for images **(D–F)**.

The selected regions in which we searched VENs had the sub-laminar organization of layer V into both, a superficial (Va) and a deep (Vb) portion. Layer V had the presence of pyramidal cells whose density decreased going deeper (Vb); on the contrary, their soma area increased, which constituted a criterion for its subdivision (Öngür et al., 2003; Buriticá and Pimienta, 2007). Figures 1B, 2B show the distinctive features of layer V, as well as its limits with respect to the inner granular layer (IV) and the multiform layer (VI).

Although VENs were present in all the postmortem subjects, they were very scarce and were not present in all the tissue sections nor were evenly distributed in the crest and walls of the gyrus of each section. For this reason, it was possible to establish the total number of VENs in all the processed tissue sections. To establish the VEN/pyramidal ratio in those sections of tissue where VEN was found, we counted the total number of pyramidal cells and VENs within grids (described below) located on sublayer Vb. To establish possible differences in the distribution of VENs between portions of the same gyrus, we placed two-dimensional grids measuring 1500 μm long × 300 μm thick along the layer Vb (previously, the average thickness of sublayer Vb of the medial BA10 was established in all the analyzed tissue sections, and thus, the thickness of the grid covering the entire sublayer was determined) both on the crest and on the walls of each tissue section where VENs were found (see Figure 1C). Within each grid, we counted all the pyramidal cells and VENs that were present.

Within these grids we also chose pyramidal neurons whose soma area was measured to compare with the VENs soma area. The fusiform cells of layer VI whose soma area was compared with pyramidal cells and VENs were located just below the grids. This procedure was performed for neurons that were overlap-free and had no defects that could have affected their measurement. The neuronal soma area was determined by delineating the perimeter of the soma in microphotographs of magnification 40× properly calibrated, and the software gave us the measurement of the bi-dimensional area of the soma in μm^2^. We wanted to determine the soma area of all the VENs found, and of 10 pyramidal and three fusiform cells for each VEN; however, we could not determine the soma area of all the expected neurons because some of them were overlapped with another neuron which prevented us to clearly see the boundaries between one neuron and another. In total, we established the soma area of 69 VENs, 570 pyramidal and 176 fusiform cells. These counts and measurements were made with the assistance of the Sigma Scan Pro five program (SPSS Science 2000^®^).

### Statistical Analysis

In Table 1, data are expressed as the average (SE) of the quantification of VENs and pyramidal cells of each subject in the tissue sections where VENs were found. In Table 2 data are expressed as the average (SD) of the quantification of VENs and pyramidal cells of each hemisphere in the tissue sections where VENs were found. The VEN/pyramidal ratio will be shown by the expression A:B, where A corresponds to the number of VENs and B corresponds to the number of pyramidal cells found for each VEN. The neuronal soma area will be expressed in μm^2^, showing the average value (SD). The analysis of the data was performed with the GraphPad 5.0 software. Data were analyzed considering statistically significant differences with a minimum significance level of *p* < 0.05. The VEN/pyramidal ratio between crests and walls of the gyri was compared using a student’s *t*-test. The VEN/pyramidal ratio between hemispheres was compared using a student’s *t*-test. The soma area of VENs with respect to pyramidal and fusiform cells was analyzed using one-way ANOVA; and the soma area of the three types of neurons between crest and walls of the gyrus was analyzed using two-way ANOVA. For both cases, comparisons were made intra- and inter-hemispherically.

**Table 1:** Number of von Economo neurons (VENs) per subject in the human medial frontopolar cortex.

Subject	Number of sections	Number of VENs		Number of pyramidal cells		Ratio	
	A/B	CREST	WALL	CREST	WALL	CREST	WALL
1	8/12	8.40 (3.59)	4.33 (0.88)	328.40 (30.59)	319.00 (34.87)	1:39	1:74
2	4/12	1.50 (0.50)	1.00*	354.50 (47.50)	398.00	1:236	1:398
3	5/9	2.00 (1.00)	1.00 (0.00)	446.50 (23.50)	464.00 (55.77)	1:223	1:447
4	7/12	2.20 (0.73)	2.00 (1.00)	304.60 (18.45)	350.00 (27.00)	1:138	1:175
5	9/12	1.20 (0.20)	1.25 (0.25)	276.80 (8.10)	343.30 (26.12)	1:231	1:275

The table shows the average number of VENs (SE), the average number of pyramidal cells (SE), and the VENs/Pyramidal cells ratio from all the tissue sections with VENs in each sampled subject. The counting grids (1500 μm × 300 μm) were positioned in those sectors where VENs were observed on sublayer Vb, both on the crests and on the walls of the gyri of human medial BA10. A/B: A corresponds to the number of tissue sections with VENs, and B to the number of sampled tissue sections. *Only one of the analyzed tissue sections had VENs in it. For this reason, no dispersion measure is presented. The other data show the number of neurons found in more than one tissue section.

**Table 2:** VEN/pyramidal cells ratio between portions of the gyri and between hemispheres of the human medial frontopolar cortex.

	Hemisphere	Number of VENs	Number of pyramidal cells	Ratio
TOTAL	R	3.68 (0.97)	339.60 (17.67)	1:92
	L	1.10 (0.10)*	350.00 (16.30)	1:318*
WALL	R	2.30 (0.54)	369.40 (28.51)	1:161
	L	1.00 (0.00)*	375.30 (13.59)	1:375*
CREST	R	4.83 (1.69)	314.80 (20.37)	1:65
	L	1.14 (0.14)*	339.10 (21.85)	1:297*

R, Right hemisphere; L, Left hemisphere; Ratio, VENs/Pyramidal cells proportion. The table shows the average number of VENs (SE), the average number of pyramidal cells (SE), and the VENs/Pyramidal cells ratio from all the tissue sections with VENs in each hemisphere, both on the crests and on the walls of the gyrus of human medial BA10. *Statistically significant interhemispheric differences both in the average number of VENs and in the VEN/pyramidal ratio (*p* < 0.0001 and *p* < 0.05 respectively). These significant differences are preserved by discriminating between the portions of the gyrus (crest and walls) of each hemisphere (*p* < 0.0001 and *p* < 0.05 respectively).

## Results

Considering the search pattern of the VENs described in the “Materials and Methods section” (observation along sublayer Vb) as well as the morphological characteristics described in previous studies, in the present study we report for the first time the presence of VENs in sublayer Vb on the medial surface of the human frontopolar cortex (BA10; see Figures 2A–C). VENs were observed in both hemispheres of all the subjects analyzed (see Tables 1, 2); however, these cells were not present in all the tissue sections. In the sections where they were found, VENs were observed on the crest and/or on the walls of the gyrus (see Table 1), being more abundant in the first of these sectors (*p* < 0.05). The observed VENs exhibit the elongated bodies with a single basal process and an apical process extending symmetrically. The anti-NeuN antibody labeling showed low intensity in the soma, contrasting with the high intensity in the clearly distinguishable nucleus (Figures 2C,D).

Inside the counting grids, we found 92 neurons with the VENs morphotype in all the sections analyzed belonging to all subjects sampled. However, the total number of VENs per tissue section and/or per subject was very variable, as can be seen in Table 1. There were two subjects with atypical quantities of VENs: subject 1 with an average of 8.40 (3.59) on the crests and 4.33 (0.88) on the walls, and subject 2 with a single VEN on all the walls of the observed gyrus. In this way, subject one presented 59.78% and subject 2 presented 4.35% of all VENs found in the study. The remaining 35.87% of VENs were distributed homogeneously among subjects 3, 4 and 5.

Although up to 21 VENs were found in a counting grid (from subject 1), these neurons were scattered and did not form clusters. The total number of pyramidal cells quantified in the counting grids where VENs were found was 10971. The VEN/pyramidal ratio considering the totals of each neuronal type was 1:119, that is, VENs corresponded to 0.84% of the pyramidal cells of sublayer Vb on the medial surface of the human frontopolar cortex only in those sectors where VENs were found (see Tables Table 2). VENs were always more abundant in the crests than in the walls of the gyri (see Tables 1, 2). Furthermore, we found 66 VENs on the crests and 26 on the walls in all the analyzed tissue sections (see Table 2), resulting in a VEN/pyramidal ratio that ranged between 1:39 and 1:236 on the crests, and between 1:74 and 1:447 on the walls (see Table 1). There were no statistically significant differences in the number of pyramidal cells between the crests and the walls of the gyri (*p* > 0.05). However, the VEN/pyramidal ratio differed statistically between crest and wall of the gyrus (*p* < 0.05).

Inside the counting grids, we found 81 von Economo neurons in the right hemisphere and 11 in the left hemisphere. The VEN/pyramidal ratio was 1:92 and 1:318, respectively (see Table 2). The difference in the average number of VENs and in the VEN/pyramidal ratio between hemispheres is statistically significant (*p* < 0.0001 and *p* < 0.05 respectively). These significant differences are preserved by discriminating between the portions of the gyrus (crest and walls) of each hemisphere (*p* < 0.0001 and *p* < 0.05 respectively). The counts of pyramidal cells between hemispheres did not show statistically significant differences (*p* > 0.05).

**Figure 3:**
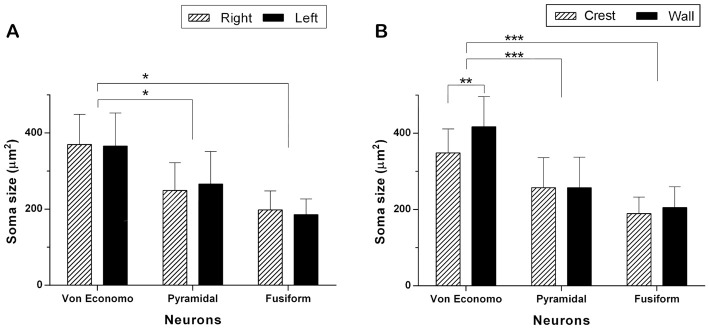
Comparison of the soma area of VENs, pyramidal and fusiform cells. **(A)** Neuronal size per hemisphere. There were no statistically significant inter-hemispheric differences in the area of VENs, pyramidal and fusiform cells. The area of the soma of VENs is greater with respect to that of pyramidal and fusiform cells (*p < 0.05). **(B)** Neuronal area per gyrus portion. There are differences in the area of VENs according to their regional location, being greater in the walls than in the crests of the gyri (**p < 0.01). VENs were larger when compared to pyramidal and fusiform cells, in both portions of the gyri (***p < 0.001). In the latter, there were no differences related to their location along a gyrus.

With respect to the area of the neuronal soma, in VENs, the average of this item was 369.29 μm^2^ (79.55 μm^2^). In the case of pyramidal cells (see Figure 2E), the average of their somatic area was 257.11 μm^2^ (79.50 μm^2^) and that of the fusiform cells of layer VI (see Figure 2F) was 196.01 μm^2^ (48.57 μm^2^). On average, VENs were 1.43 times (43.89%) larger than the pyramidal cells of layer Vb (*p* < 0.05), and 1.88 times (88.70%) larger than the fusiform cells of layer VI (*p* < 0.05). The soma area of VENs, pyramidal and fusiform neurons did not show significant differences between hemispheres (see Figure 3A). The average area of VENs in the crests was 348.40 μm^2^ (63.01 μm^2^) and 417.10 μm^2^ (93.45 μm^2^) in the walls, i.e., 19.7% larger in the latter (*p* < 0.01). The soma area of pyramidal and fusiform cells, between crest and walls of the gyrus, did not present statistically significant differences (see Figure 3B).

## Discussion

This is the first report of VENs on the medial surface of the frontopolar cortex in humans. We had previously made a search for these cells in the frontal pole, specifically on the lateral surface, with negative results (Fajardo et al., 2008). In the present study we carried out the search of VENs specifically on the medial surface of BA10. Its location in this region was also observed in sublayer Vb, as has been reported predominantly in the literature (Nimchinsky et al., 1995; Watson et al., 2006; Fajardo et al., 2008; Allman et al., 2011). In general, the morphological characteristics (shape of the soma, number and shape of its proximal processes, laminar location) of the VENs found in the present study are similar to those previously described in other human cortical regions (frontoinsular cortex, anterior cingulate region and dorsomedial BA9); however, the quantity and soma area vary.

According to previous reports, the VEN/pyramidal proportion vary from one cortical region to another ranging from 0.5% to 13% (Nimchinsky et al., 1999; Fajardo et al., 2008; Raghanti et al., 2015). In the present study, the absolute number of VENs and the VEN/pyramidal cell ratio was considerably lower than in those studies. Although we found VENs in both hemispheres of all the subjects, their presence was not continuous throughout the entire sublayer Vb or from one tissue section to another. It is important to consider that this data corresponds to the quantification made deliberately in the sectors where VENs were found, so that this value could be even lower if the total extension of sublayer Vb of the medial BA10 had been analyzed. To overcome this limitation, future work should consider the acquisition of a greater number of tissue sections. Moreover, having a representative sample of the entire cortical area under study will make it possible to apply stereological methods for volumetric analysis. More studies of medial BA10 are also required, both with neurotypical and with pathological populations to look for explanations regarding the atypical data on the number of VENs found in the present study.

Considering that our results and those reported in the frontal region of the insula, the anterior cingulate cortex and dorsomedial BA9, it is possible to suggest that in humans, VENs decrease in the postero-anterior and ventro-dorsal direction, as in that they approach the frontal pole or the dorsomedial convexity of the frontal lobe. Of course, this interpretation needs to be validated in the light of more evidence, for example, by exploring other cortical regions of the medial frontal lobe such as BA25 and BA32.

Given the small size of the tissue sample obtained for the present study, there was a lack of data regarding the presence and distribution of VENs in the depth of the sulci. We found that the VEN/pyramidal ratio and the absolute number of VENs were higher in the crest than in the wall of the gyri (see Tables 1, 2). Similar results have been previously reported (Raghanti et al., 2015), so that the evidence obtained in the present study could support the hypothesis that VENs correspond to a morphological variant of pyramidal cells located in the deep layers of the gyri after of the mechanical pressure that is present in the phenomenon of the gyrification (Van Essen, 1997; Pillay and Manger, 2007; Nie et al., 2012).

We also found that the VEN/pyramidal ratio and the absolute number of VENs were higher in the right hemisphere than in the left counterpart (see Table 2). Observations like these have already been made, both in humans and in other species (Butti et al., 2009; Allman et al., 2011; Evrard et al., 2012). It has been proposed that the greater presence of VENs in the right hemisphere of fronto-insular cortex could be related to the triggering of responses of the sympathetic division of the autonomic nervous system (Allman et al., 2011). This functional relationship could also be considered for our findings in the medial frontopolar cortex due to the connections that this cortical region presents with the hypothalamus and the periaqueductal gray matter (Rempel-Clower and Barbas, 1998; Öngür and Price, 2000). However, it would be hasty to make functional conjectures about a cell type so scarce in the studied cortical region.

On the other hand, the connections of the medial BA10 with other cortical regions in which the presence of VENs has been reported have been associated with processes such as intuitive assessment and generation of rapid responses in social contexts (Baron-Cohen et al., 1999; Butti et al., 2013; Cauda et al., 2013). These functions are affected in pathologies such as autism, schizophrenia and frontotemporal dementia, in which these cells appear to be selectively vulnerable (Allman et al., 2005; Krause et al., 2017; Yang et al., 2017; Gefen et al., 2018). According to the cortical organization pattern of the frontal pole on the medial surface, this region would send projections from the infragranular layers to the supragranular layers of other cortical sectors that have higher levels of hierarchical organization such as the dorsolateral prefrontal cortex (Barbas and Rempel-Clower, 1997). Although it is possible to assume that the axons of VENs share this same pattern of cortico-cortical connections of the pyramidal neurons of sublayer Vb, given the scarce presence of them it is difficult to assign them a specific functional role related to the mental functions attributed to the medial BA10, such as prospective memory, attention control, theory of mind and metacognition (Gusnard et al., 2001; Burgess et al., 2007a,b).

In the human medial frontopolar cortex, the soma area of VENs is larger than that of pyramidal cells of layer Vb, and that of fusiform neurons of layer VI. However, the difference in soma area between VENs and pyramidal cells found in the medial BA10 is less significant than the difference reported in other human cortical regions (Nimchinsky et al., 1999; Fajardo et al., 2008). In our study we found that the soma of VENs in the walls is larger than in the crest of the gyri. This is a novel finding because there are no previous reports in the literature that compare the soma area of the VENs between the crest and the wall of a gyrus. If the soma of VENs is reduced in some cortical areas such as the medial frontopolar cortex and is almost the same to that of the pyramidal cells of layer V, perhaps this morphological peculiarity manages to hide them in cortical regions where they have not been found and to explain their circumscribed location in humans.

In human medial BA10, data such as the observed small number of VENs and the small differences in the soma area with respect to the pyramidal cells, would suggest that in addition to being a specific cell type, the morphology of VENs might be the product of mechanical forces experienced during the gyrification of cerebral cortex (Van Essen, 1997). This idea could be explored from a histological analysis of different biochemical markers in the infragranular layers, as was performed by Stimpson et al. (2011), and thus it could be established if the VENs of the medial BA10 only have morphological differences with respect to the pyramidal cells of their same layer or if there are other characteristics that distinguish them.

## Author Contributions

CAG-A and EB conducted the experiments and carried out the data analysis. CAG-A, MIE, HP and EB designed the experiments. CAG-A, MIE, MFC, HP and EB wrote the manuscript.

## Conflict of Interest Statement

The authors declare that the research was conducted in the absence of any commercial or financial relationships that could be construed as a potential conflict of interest. The reviewer MV and handling Editor declared their shared affiliation.
